# Basic Properties and Development Status of Aluminum Adjuvants Used for Vaccines

**DOI:** 10.3390/vaccines12101187

**Published:** 2024-10-18

**Authors:** Jingyang Lan, Disong Feng, Xueshan He, Qianru Zhang, Rong Zhang

**Affiliations:** School of Life Science and Bio-Pharmaceutics, Shenyang Pharmaceutical University, Shenyang 117004, China; lan13434124762022@163.com (J.L.); fengdisongii@163.com (D.F.); zqr18834895240@163.com (Q.Z.)

**Keywords:** aluminum adjuvants, vaccines, physical and chemical properties, mechanisms

## Abstract

Background: Aluminum adjuvants, renowned for their safety and efficacy, act as excellent adsorbents and vaccine immunogen enhancers, significantly contributing to innate, endogenous, and humoral immunity. An ideal adjuvant not only boosts the immune response but also ensures optimal protective immunity. Aluminum adjuvants are the most widely used vaccine adjuvants and have played a crucial role in both the prevention of existing diseases and the development of new vaccines. With the increasing emergence of new vaccines, traditional immune adjuvants are continually being researched and upgraded. The future of vaccine development lies in the exploration and integration of novel adjuvant technologies that surpass the capabilities of traditional aluminum adjuvants. One promising direction is the incorporation of nanoparticles, which offer precise delivery and controlled release of antigens, thereby enhancing the overall immune response. Conclusions: This review summarizes the types, mechanisms, manufacturers, patents, advantages, disadvantages, and future prospects of aluminum adjuvants. Although aluminum adjuvants have certain limitations, their contribution to enhancing vaccine immunity is significant and cannot be ignored. Future research should continue to explore their mechanisms of action and address potential adverse reactions to achieve improved vaccine efficacy.

## 1. Introduction

Vaccine adjuvants are a class of non-antigenic substances that nonspecifically modify or enhance the body’s specific immune response to antigens [1]. These adjuvants can induce long-term and efficient specific immune responses, thereby improving the efficacy of vaccines and extending the duration of immune protection [2]. Additionally, vaccine adjuvants can reduce the amount of antigen required, lower production costs [3], and decrease the number of clinical immunizations needed. Due to the limited availability of adjuvant materials, many vaccines were created using live-attenuated viruses (such as the MMR and chickenpox vaccines) to leverage their natural adjuvant properties. However, these vaccines pose a risk to immunocompromised individuals. Additionally, numerous vaccines were developed with inactivated pathogens without adjuvants (like the seasonal flu and polio vaccines), which often struggle to achieve sufficient efficacy on their own. Moreover, with the introduction of recombinant protein antigens in the 1980s, which are less immunogenic than live-attenuated or whole killed antigens, the need for advancements in adjuvant technology became increasingly evident [4].

Aluminum adjuvants have been widely recognized [5], and they are used in veterinary and human vaccines because they are relatively safe to use. Since Glenny’s [6] discovery in 1926 that diphtheria toxoid (DT) suspension has greater antigenicity than the toxoid itself, aluminum adjuvants have been widely used in both human and animal vaccine preparations. The introduction of aluminum hydroxide adjuvant in 1939 facilitated the commercialization of aluminum adjuvants, which have gradually become a crucial means of enhancing the immune response to vaccines [7]. However, with the advancement of vaccine research and the extensive use of aluminum adjuvants [8], the requirements for their types and characteristics have also increased. Aluminum adjuvants have limitations in their physical and chemical properties, unclear mechanisms of immune response, insufficient cellular immunity, and suboptimal enhancement of immune responses to small molecule antigens. Additionally, they can cause local irritation and adverse reactions, significantly limiting their broad application in vaccines [9].

Non-aluminum adjuvants have shown greater effectiveness in triggering cell-mediated immune responses, independent of aluminum-based formulations. MF59, a pioneering non-aluminum adjuvant, was one of the first to be approved for use in human vaccines, specifically for influenza, in 1997 [10]. Additionally, numerous other non-aluminum adjuvants are currently undergoing various stages of research and development. Given the challenges in achieving key technological breakthroughs and improving the performance of traditional aluminum adjuvants, the application of molecular biology techniques has emerged as a new research hotspot [11]. A recent study has revealed a nano-aluminum adjuvant exhibiting superior uniformity and increased phagocytosis of the encapsulated antigen particles by macrophages [12], which reduces the required dosage of aluminum adjuvant and minimizes vaccine-related adverse reactions.

Despite its use as a vaccine adjuvant for over 80 years, the precise mechanism of an aluminum adjuvant remains somewhat unclear [13]. In recent years, the application of aluminum adjuvants in vaccines has garnered widespread attention, prompting many researchers to conduct in-depth discussions and summaries on this topic [14]. Laera’s review [15] focuses on how aluminum adjuvants selectively stimulate Th2 immune responses and their activation mechanisms on dendritic cells (DCs). His findings indicate that aluminum adjuvants can effectively activate dendritic cells by inducing cytokine secretion and promoting antigen presentation, thereby influencing subsequent immune responses. This paper reviews the types, mechanisms, and current development status of aluminum adjuvants both domestically and internationally, aiming to provide a reference for further research and the rational application of aluminum adjuvants.

## 2. Types of Aluminum Adjuvants

**Figure 1:**
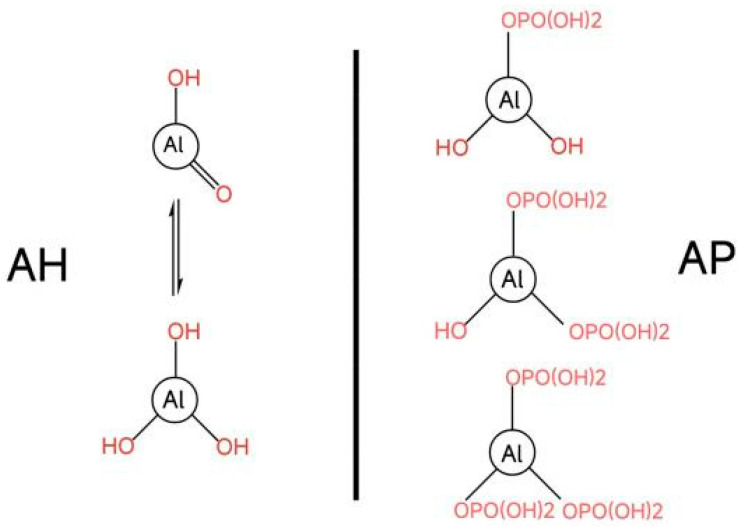
AH and AP molecules. On the **left**, a depiction of the two variants of AH, and on the **right**, the varying levels of phosphate group substitution in the AP molecule.

At present, mainstream aluminum adjuvants include the following three categories: aluminum hydroxide [Al(O)OH], aluminum phosphate [Al(OH)_x_(PO4)_y_], and aluminum potassium sulfate [KAl(SO_4_)_2_·12H_2_O] [16]. Aluminum hydroxide and aluminum phosphate are among the most commonly used adjuvants in vaccines [17]. Aluminum hydroxide is a layered crystal with only a hydroxyl group on the surface. The isoelectric point (pI) is 11.4, and the molecule carries no net charge. Its hydroxyl group can either donate or accept protons, contributing to its behavior in different pH environments. In a neutral solution at pH 7.4, it typically exists in a cationic form, carrying a positive charge. It is an effective adsorbent for anionic antigens and can efficiently adsorb acidic protein vaccines. It exists as fibrous particles with dimensions of 4.5 nm × 2.2 nm × 10 nm [18]. These particles accumulate in a loose form, ranging in size from 1 to 10 μm [19]. The surface area can reach up to 510 m^2^/g, with antigen binding occurring only on the outer layer of the adjuvant particles. The aluminum phosphate adjuvant is a hydroxyl aluminum phosphate complex with an amorphous structure [20]. Its primary particles are disk-shaped with a diameter of 50 nm [21], which subsequently form loose aggregates approximately 3 μm in size. The displacement degree of the phosphate group to the hydroxyl group depends on the reactant and precipitation conditions. Its isoelectric point ranges between 4 and 7, exhibiting an anionic form in pH 7.4 solutions, making it an effective adsorbent for cationic antigens (Figure 1). 

## 3. Mechanism of Immune Enhancement in Aluminum Adjuvants for Vaccines

At present, the specific mechanism of immune enhancement in aluminum adjuvant for vaccines is not clear [22]. Many scholars have discussed the mechanism and carried out a lot of studies and experiments [23], among which the more mainstream mechanism explanations are as detailed below.

### 3.1. Repository Effect

**Figure 2:**
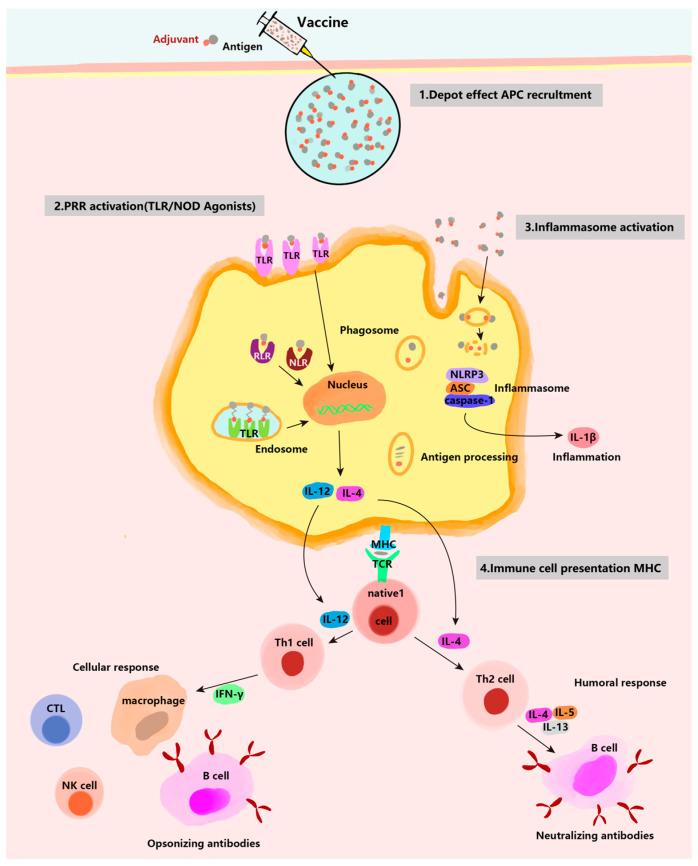
Several mechanisms of action of adjuvants: (1) depot effect; (2) PRR activation; (3) NLRP3 inflammasome activation; (4) immune cell presentation.

Following adsorption, antigens accumulate on and within the aluminum-hydroxide-based adjuvant particles, preserving their physical and chemical properties. These adjuvant particles present the stored antigens to immune cells, facilitating prolonged interactions between the antigens and immune cells to trigger immune responses. This process is known as the “repository effect” or “depot effect” [24]. The repository effect suggests that after adsorption by the aluminum adjuvant, antigens are highly aggregated at the injection site and within the tissue, allowing for prolonged and effective presentation to immune cells. This enhances immune cell contact with the antigen, thereby effectively inducing an immune response (Figure 2). Glenny [25] found that the adsorption of antigens by aluminum adjuvant reduces antigen loss at the injection site, facilitates the slow release of antigens, prolongs antigen retention time in the body, and continuously stimulates the immune system. Experiments have confirmed that some antigens can persist at the injection site of aluminum-adsorbed diphtheria toxoid for at least 7 weeks post-injection.

In recent years, the study of aluminum adjuvants has deepened our understanding of the antigen storage effect, leading to some controversial new findings that challenge earlier theories. The repository effect was first questioned for its mechanism as far back as 1950, when Holt [26] proposed and confirmed that the inoculation of precipitated aluminum diphtheria toxoid did not interfere with the immune response to diphtheria toxoid production after seven days of elimination from the injection site. However, after 14 days, the infiltration and reinoculation at the same injection site in guinea pigs enhanced the immune response compared to animals with intact injection sites, suggesting that antigen storage was not suitable for the immune system [27]. Aluminum phosphate adjuvants can enhance the immune response to vaccines [28], unlike the latest synthesis and secretion of antigens, aluminum salt adsorption, and the adsorption of interstitial proteins with high affinity for aluminum salts and antigens, which do not always require aluminum adjuvants. As long as the antigen concentration is sufficient to allow dendritic cells to absorb the antigen, aluminum adjuvants may enhance vaccine responses and produce direct or indirect effects on antigen-involved cells.

### 3.2. Immunostimulatory Response

After being injected into the body, an aluminum adjuvant can induce an endogenous immune response and attract a substantial number of inflammatory cells at the injection site. Recent evidence suggests that the inflammatory response plays a crucial role in the immune response to aluminum adjuvant vaccines [29]. To better understand this process, aluminum adjuvant vaccines were administered to unvaccinated or previously immunized mice, and the injection sites were characterized at different time points post-immunization to analyze the primary and secondary inflammatory responses. From 2 to 6 h after inoculation, inflammatory cells appeared at the injection site, initially dominated by neutrophils, followed by macrophages. Over time, the number of eosinophils and MHC II+ cells increased, while that of neutrophils decreased after day 2.

Macrophages can extensively phagocytose aluminum adjuvant particles, leading to faster and stronger recruitment of eosinophils, macrophages, and antigen-presenting cells at the injection site in secondary immunized mice [30]. However, some studies have shown that macrophages at the injection site rapidly disappear, sensing the imbalance of homeostasis and quickly releasing cytokines and chemokines. This activates the immune system and recruits endogenous immune cells. The differing findings may be attributed to the choice of different injection routes and adjuvant types. IL-5 and histamine, released by macrophages and mast cells, attract eosinophils to migrate to the injection site [31].

Investigations have revealed that the administration of aluminum-based adjuvants leads to the recruitment of neutrophils to the injection site, facilitated by mast cells [32]. These neutrophils are capable of releasing cytokines such as IL-1β, IL-5, and chemokine ligand 2, which, in turn, modulate the expression and secretion profiles of activated normal T cells. Consequently, this results in the attraction of neutrophils and eosinophils to the site of injection. Beyond their direct role in pathogen elimination, these cells contribute to the establishment of acquired immunity [33]. There is substantial evidence suggesting that aluminum adjuvants can indirectly activate antigen-presenting cells and human peripheral blood mononuclear cells. Notably, the use of aluminum hydroxide-adsorbed diphtheria toxoids has been correlated with the induction of IL-1 secretion. Furthermore, it has been demonstrated that employing aluminum gel particles (with a diameter of 10 μm), as opposed to soluble antigen alone, enhances phagocytic activity [34].

Rimaniol [35] studied the interaction between aluminum hydroxide adjuvant and macrophages through in vitro tests. The study found that macrophages undergo changes in cell phenotype and function, becoming more similar to myeloid dendritic cells, which can induce an MHC-class II antigen-specific immune memory response. Aluminum hydroxide was shown to enhance MHC expression [36], and peripheral blood monocytes, when stimulated with several composite molecules, exhibited increased mRNA expression of IL-4 while inhibiting the elevation of IL-1, TNF, and IL-6. Most monocytes, distinct from DCs, displayed indirect morphological changes and enhanced CD83 expression, a marker of mature DCs. The neutralization of IL-4, depletion of CD4 T lymphocytes, and increased monocyte MHC expression suggest that the effect of the aluminum adjuvant on these cells is indirect [37].

### 3.3. Pro-Phagocytic Effect

Aluminum adjuvant can enhance the antigen uptake capacity of monocytes and dendritic cells in macrophages and peripheral blood. Complexes containing aluminum adjuvants promote the activation and maturation of antigen-presenting cells [38], and studies have shown that they activate monocytes to differentiate into dendritic cells, thereby enhancing their antigen uptake capacity. Aluminum adjuvant vaccines and these mature antigen-presenting macrophages are particularly important in establishing memory responses and vaccination mechanisms that lead to long-term protection. They directly act on macrophages, promoting their maturation. The aluminum adjuvant also assists in the transfer of antigen-presenting cells within lymphoid tissue. Following subcutaneous or intramuscular injection, these cells continue to migrate to distant lymph nodes and the spleen. Additionally, dendritic cells loaded with aluminum adjuvant vaccines move to the blood through the thoracic duct and circulate to various organs. Aluminum adjuvant can also induce IL-1β and IL-18 [39], promoting the differentiation of naive CD4+ T cells into Th2 cells and enhancing antibody production.

### 3.4. The NLRP 3 Inflammatory Response

It has also been found that the adjuvant action of aluminum requires the involvement of the inflammasome NLRP3 [40], which promotes the secretion of high levels of the pro-inflammatory factors interleukin-1β (IL-1β) and IL-18 by macrophages [41]. Different research groups have provided varying answers regarding whether the adjuvant activity of aluminum depends on the formation of the NLRP3 inflammasome. For instance, Eisenbarth [42] and Li [43] found that the NLRP3 inflammasome induced by aluminum adjuvant is necessary for its immunomodulatory activity. However, granular substances such as aluminum adjuvant can also induce the production of pro-inflammatory factors through an NLRP3-independent pathway [44]. By immunizing NLRP3-deficient mice with aluminum adjuvant, it was observed that the deficiency of NLRP3 had no significant effect on T and B cell responses. Therefore, the role of the NLRP3 pathway in adjuvant-stimulated immunity requires further investigation [45] (Figure 2).

In conclusion, aluminum adjuvants enhance the immune response through multiple mechanisms, which require ongoing exploration and experimentation for a comprehensive understanding. Therefore, a deeper understanding of the mechanisms, advantages, and disadvantages of aluminum salt adjuvants will aid in the development of safer and more effective vaccine adjuvants, ultimately improving protection for those who receive the vaccines.

## 4. Interaction Relationship Interaction with Antigens

The interaction between the antigen and aluminum adjuvant primarily relies on electrostatic attraction, hydrophobic interactions, and ligand exchange [46]. When antigens and adjuvants carry opposite charges, electrostatic attraction becomes significant. Antigens adsorbed through electrostatic forces can be swiftly released from aluminum adjuvants in tissue fluid. The strongest bond between antigens and aluminum adjuvants occurs through ligand exchange [47]. When antigens possess phosphate groups, these groups interact with the aluminum by exchanging with the hydroxyl groups on the adjuvant’s surface, causing conformational changes in the proteins. This facilitates the formation of inner sphere complexes, also known as ligand exchanges [48]. Antigens bound to aluminum adjuvants through ligand exchange are released gradually in tissue fluid. However, factors such as pH, temperature, buffer strength, ion composition, and the physical properties of proteins also influence the adsorption of proteins onto aluminum adjuvants. These variables determine whether aluminum salts can be effectively used as adjuvants in certain vaccines.

Commonly used aluminum-containing additives tend to aggregate in water. Both adjuvants form colloidal particles ranging from several micrometers in size when dispersed in water, and their effectiveness can be influenced by the physical and chemical properties of the particles [49]. Aluminum-salt-based adjuvants enhance the uptake of the antigen [50] and improve the development of immune memory after vaccination through an enhanced humoral immune response [51]. Aluminum salts adsorb antigens primarily through electrostatic interactions or ligand binding [52]. Antigen adsorption can affect antigen stability [53] and the immune response, as well as the cellular uptake and presentation of the antigen [54]. The adsorption degree of vaccine antigen is determined during the batch release of vaccines containing aluminum salt adjuvants. The adsorption capacity is influenced by various factors, such as the charge and size of the adjuvant and antigen. Moreover, the surface area of the aluminum-salt-based adjuvant is crucial for protein adsorption [55].

In the case of aluminum adjuvant-coated antigens, the ratio of surface area to volume increases, resulting in a relative increase in surface area, which means that they are able to provide more response sites. Beyond influencing antigen adsorption capacity, the particle size of an adjuvant is a critical parameter in shaping the immune response [56]. The cellular uptake of granules is often size-dependent, with distinct uptake pathways for small and large particles, which can impact the resulting immune response [57]. Studies have shown that specific antibody responses are stronger and more sustained following subcutaneous immunization with aluminum hydroxide nanoparticles compared to aluminum hydroxide microparticles. These effects are attributed to enhanced antigen adsorption by antigen-presenting cells (APCs) and improved absorption of adjuvant–antigen complexes when using nanoparticles [58]. In addition to particle size, the shape and crystallinity of aluminum adjuvants have been demonstrated to significantly influence the duration and intensity of immune responses. The research conducted by Bingbing Sun et al. [59] has corroborated that these two physical properties are pivotal in activating the NLRP3 inflammasome, which subsequently leads to the production of IL-1β in dendritic cells and enhances the ovalbumin (OVA)-specific immune response in mice.

Regarding chemical properties, the surface charge of aluminum adjuvants exerts a substantial influence on their antigen adsorption capacity. It is generally observed that aluminum hydroxide adjuvants are more effective with acidic proteins, whereas aluminum phosphate adjuvants exhibit greater efficacy with alkaline proteins. Acidic antigens with an isoelectric point of 5–6 are readily adsorbed onto the boehmite form of aluminum hydroxide gel. However, a shift in the buffer system to a phosphate-based environment results in a diminished adsorption capacity. Conversely, alkaline proteins with an isoelectric point of approximately 12 display an opposite pattern of adsorption [60]. These observations underscore the importance of optimizing the physicochemical properties of aluminum adjuvants to enhance their immunostimulatory potential. Factors such as pH, temperature, buffer strength, ionic composition, and the physical properties of proteins also influence the adsorption of proteins. These variables can limit the effectiveness of aluminum salts as adjuvants for certain vaccines [61]. Additionally, conditions such as low ionic strength, fewer phosphate groups, and minimal impurities can enhance the adsorption of antigens onto the surface of aluminum adjuvants.

## 5. Advantages and Disadvantages of Aluminum Adjuvants

The advantages of aluminum adjuvants include their effective adsorption of soluble antigens onto their surface, which helps concentrate the antigen and reduce the required injection dose [62]. Additionally, aluminum adjuvants are low-cost, easy to use, and non-toxic. As a result, they are the most widely used adjuvants in veterinary biological products and are the only adjuvants approved by the FDA for human vaccines.

Adjuvants such as aluminum salts are designed to enhance the antibody response of vaccines, thereby increasing their effectiveness. However, it is essential to acknowledge the controversies surrounding these adjuvants, particularly in the context of hepatitis B and HPV vaccines [63], which have been associated with autoimmune and neurological diseases [64]. There are inherent issues with aluminum adjuvants; aluminum salts are often not effective adjuvants, especially in inducing cellular immune responses. Granulomas are often produced when aluminum adjuvants pass through the route of subcutaneous or intrasdermal rather than intramuscular injections [65]. To date, all papers addressing the deleterious effects of aluminum have identified two primary mechanisms responsible for aluminum toxicity: direct aluminum toxicity and aluminum-induced cellular damage via oxidative metabolism. Based on fundamental principles of biochemistry and inorganic chemistry, it is proposed that aluminum significantly disrupts iron homeostasis, ultimately leading to iron-mediated cellular injury [66]. Another side effect is increased IgE production, metamorphosis and possible neurotoxicity.

Any adjuvant can have side effects. In the case of aluminum hydroxide adjuvant combined with antigen, there may be the following injection site reactions [67]: swelling, erythema, pain, induration, and ecchymosis. In addition, there is a minimal chance of systemic reaction, including fever, fatigue, myalgia, diarrhea, dizziness, vomiting, etc., but the above are mild and temporary local and systemic reactions. The symptoms often improve within 72 h of injection and may disappear within 28 days. There are upper limits on the aluminum adjuvant content used for human vaccines, which may affect the efficacy of certain vaccines. Bresson [68] reported that the H5N1 influenza vaccine enhanced the immune response with aluminum adjuvant at high doses (30 μg), but the adjuvant for low doses (7.5 μg and 15 μg) was able to induce an immune response similar to the 15-microgram dose; this suggests that the immune response is not always linearly enhanced with increasing the dose. In addition, high concentrations of aluminum adjuvant may also have toxic effects on phagocytes [69].

Treanor [70] conducted a multicenter, double-blind, two-stage study involving 451 healthy adults (aged 18 to 64) who were randomly assigned to receive two intramuscular injections of the H5N1 subunit influenza vaccine containing 90, 45, 15, and 7.5 micrograms of hemagglutinin antigen or placebo. The study tracked the safety data of each group for up to 56 days- The local side effects in the adjuvant group were generally increased compared with the placebo group, but there was no significant difference between the two groups regarding systemic side effects [71]. Boretti’s [72] manuscript explores the link between aluminum adjuvants in vaccines and autism spectrum disorder (ASD). Aluminum is known to be neurotoxic. Infants who have received vaccines containing AlAd exhibit a higher incidence of ASD. Behavioral changes are observed in mice following Al injections. Patients with ASD have elevated levels of Al in their brains. Consequently, aluminum adjuvants (AlAds) are considered a causative factor in ASD [73]. Numerous studies have documented elevated aluminum concentrations in the brains of individuals with Autism Spectrum Disorder (ASD) as compared to neurotypical controls, implying a possible contribution of aluminum to the etiology of ASD [74]. Furthermore, experimental animal models have demonstrated that aluminum administration can elicit behavioral alterations in mice, reinforcing the hypothesis that exposure to aluminum may be associated with the onset of autism-like symptoms [75]. These findings warrant further investigation into the relationship between aluminum exposure and the development of ASD. While AlAds’ immune-enhancing properties have justified their use in vaccines, the safety of vaccinated individuals has not been adequately addressed. The mechanisms by which an AlAd operates and the pharmacodynamics of AlAds in vaccines remain poorly understood. Multiple lines of evidence suggest a connection between aluminum adjuvants in vaccines and autism spectrum disorder. However, the relationship between the two still needs to be further explored.

Additional pathological conditions associated with cerebral aluminum (Al) deposition are highlighted in other research. Mold’s research found that the content of aluminum in the brain tissue of multiple sclerosis (MS) patients is generally higher, which may partially explain the high level of aluminum excretion in MS patients. Although it is difficult to directly associate aluminum with the pathological changes unique to MS, research suggests that aluminum may have a promoting effect in the MS process and is associated with neurodegenerative damage [76]. The impact of aluminum on neurological diseases also includes epilepsy [77], cerebral amyloid angiopathy [78], Alzheimer’s disease [79], and cerebral palsy [80].

Gherardi et al. [81] examined the onset of myalgia and chronic fatigue syndrome post-vaccination. The enduring neurotoxic consequences of AlAd in vaccines underscore the imperative to curtail its non-essential utilization. Strategies include restricting vaccination to essential immunizations, calibrating adjuvant dosage based on the human body weight, modifying adjuvant formulations, or avoiding concurrent multiple vaccinations in infants [82].

## 6. Manufacturing and Development Status

Aluminum adjuvant is the most widely used adjuvant in human body [83]. Currently, aluminum adjuvants are used for the commercialization of human vaccines, which mainly include the following child vaccines: adsorption diphtheria–tetanus vaccine, DPT triple vaccine (DTP), no cell vaccine (DTaP), type b haemophilus vaccine (Hib), pneumonia conjugate vaccine, DPT-B haemophilus (DTP-Hib), hepatitis b vaccine–type b influemophilus (Hepb-Hib), hepatitis B vaccine (HepB), b haemophilus vaccine (PRP-OMP), etc. The adult vaccines include the following: adsorbed tetanus toxin, adsorbed tetanus–diphtheria combination vaccine, hepatitis A vaccine (HepA), Lyme disease vaccine (Lyme), anthrax vaccine (Anthrax), rabies vaccine, etc. [84]. Aluminum adjuvants are extensively incorporated into veterinary vaccines to enhance immunogenicity [85]. Such applications encompass a range of viral vaccines designed to combat diseases including avian infectious bronchitis, canine hepatitis, foot-and-mouth disease, and Newcastle disease. Additionally, these adjuvants are utilized in bacterial vaccines, such as inactivated Pasteurella vaccines, Bordetella vaccines, Clostridium botulinum vaccines, Haemophilus vaccines, and Leptospira vaccines. The use of aluminum adjuvants also extends to antiparasitic vaccines targeting helminthic infections.

Adju-Phos^®^ adjuvant is an aluminum phosphate wet gel suspension. Aluminum-based adjuvants, including Adju-Phos^®^ and Alhydrogel^®^ (aluminum hydroxide), are the most widely used type of adjuvants. Both of these products enhance Th2 antibody responses but do not significantly stimulate Th1 cellular responses. At present, the aluminum hydroxide adjuvant Alhydrogel^®^ produced in Denmark is recognized as the international public standard, and its colloidal particles are about 3.07 μm. At pH levels between 5 and 7, Alhydrogel^®^ works as an effective protein antigen-binding vaccine adjuvant. It has a low conductivity due to the absence of buffering ions and is positively charged at a neutral pH. This allows for the effective adsorption of negatively charged protein antigens [86]. The most mainstream commercial aluminum phosphate adjuvant in the world is Adjus-phos^®^, produced in Denmark. Its glue particle diameter is about 4.26 μm, and its isoelectric point range is 4.5–5.5. Aluminum phosphate maintains a certain stability under high temperature and pressure; so, it is widely used for aseptic treatment before vaccine preparation. Adju-Phos^®^ adjuvant is produced by Brenntag Biosector (Copenhagen, Denmark) [87], a leading company in the global vaccine adjuvant market with extensive experience in delivering high-quality products. Current research highlights several factors that could substantially influence its adsorption capacity. These factors include the molecular weight of protein antigens, the presence of sodium chloride, phosphate buffer, denaturing agents, and the particle size of aluminum. The Adju-Phos^®^ adjuvant’s adsorption capacity ranges from 0.4 to 0.6 mg of antigen per mg of aluminum. One notable distinction between these two adjuvants is that Adju-Phos^®^ exhibits greater solubility compared to Alhydrogel^®^ following administration [88].

The aluminum adjuvant content significantly influences the safety and immunogenicity of prophylactic vaccines. Consequently, regulatory agencies have established stringent guidelines regarding aluminum adjuvant content. The types and aluminum content of vaccines licensed in the US vary depending on the manufacturer. For instance, the DTap vaccine from GSK contains 0.6 mg of aluminum hydroxide, while the DTap vaccine from Sanofi-Pasteur contains 0.33 mg of aluminum phosphate (Table 1). Due to the critical impact of aluminum adjuvant content on the safety and immunogenicity of prophylactic vaccines, regulatory agencies have imposed strict regulations on its content.

Aluminum adjuvants are widely used in various Chinese vaccines, including inactivated vaccines, subunit vaccines, genetically engineered recombinant protein vaccines, and combination vaccines. Currently, China has multiple vaccines containing aluminum adjuvants approved. These include adsorbed diphtheria vaccine, adsorbed tetanus vaccine, adsorbed diphtheria–tetanus vaccine, adsorbed diphtheria–tetanus–pertussis (DTP) vaccine, acellular DTP vaccine, tick-borne encephalitis inactivated vaccine, hemorrhagic fever with renal syndrome inactivated vaccine, hepatitis B inactivated vaccine, and recombinant hepatitis B vaccine, among others (Table 2). These vaccines have been used in China for many years, with extensive clinical applications demonstrating their safety and efficacy. They have played a significant role in the prevention and control of infectious diseases.

At present, Chinese new aluminum adjuvant patent technology is booming with the expansion of the market and clinical demand. The new technologies developed in these patents can improve the adjuvant performance of aluminum hydroxide, enhance its cellular immune effect, and reduce the adverse reactions brought on by aluminum adjuvant. Li [89] has developed an innovative adjuvant based on aluminum hydroxide for use in foot-and-mouth disease vaccines, alongside a novel method for vaccine formulation. The invention aims to present a composite formula of the aluminum hydroxide adjuvant specifically tailored for foot-and-mouth disease, complemented by a detailed methodology for its preparation. This advancement addresses the limitations inherent in previous technological approaches, offering a potentially superior alternative for vaccine development in this context.

Zhang [90] developed a nano-aluminum adjuvant in combination with an autologous tumor vaccine by integrating the nano-aluminum adjuvant with the autologous tumor vaccine, and provided a method for preparing this nano-aluminum adjuvant. In comparative experiments with conventional aluminum adjuvant, the nano-aluminum adjuvant combined with the autologous tumor vaccine significantly inhibited the mass and volume of H22 liver cancer, and exhibited markedly higher cytotoxic activity compared to the conventional aluminum adjuvant.

Hong [91] developed a new aluminum adjuvant specifically targeting dendritic cells. This invention involved the construction of an antigen carrier system, which was a system of antigen vectors based on β-dextran particles (GPs) wrapped in an aluminum hydroxide colloidal “GP-Al” structure to enhance humoral immunity and cellular immune response. The GP-Al particles, measuring 2–4 microns in diameter, can easily load antigenic proteins through mixing, eliminating the need for complex chemical or physical manipulation. This reduces antigen loss during delivery.

Both in vitro and in vivo experiments confirmed the superior adjuvant properties of GP-Al particles as vaccine carriers. The system specifically targets APCs, significantly promoting dendritic cell maturation and cytokine secretion. It also enhances the proliferation and activation of antigen-specific CD8T cells. In animal models, GP-Al particles induce strong Th1-biased immune responses and high antibody titers, showing notable preventive and therapeutic effects on tumor growth. This demonstrates the vector system’s capability to stimulate both humoral and cellular immune responses. We believe this system can specifically target APCs and has broad applications in targeted drug delivery and expanding the use of aluminum adjuvants.

In response to the conventional crystal structure associated with aluminum hydroxide adjuvants, Chen [92] engineered an aluminum hydroxide adjuvant that augments its protein adsorption capacity through an optimized structural configuration. The invention encompasses a methodical approach to the preparation of this adjuvant, which involves elevating ion concentration, reaction temperature, and precisely modulating the reaction endpoint. This controlled transformation facilitates the transition of aluminum hydroxide from an amorphous to a crystalline state, culminating in the formation of a robust, albeit non-traditional, “bad crystal” structure that enhances the performance of the aluminum hydroxide adjuvant.

## 7. Aluminum Adjuvant Improvement and Hot Spot Direction

### 7.1. Physical Properties of the Aluminum Adjuvant

Current studies improve the performance of aluminum adjuvants by phosphorylation or adding new substances. Liu [93] and others have also confirmed that the addition of phosphate buffer to the malaria vaccine can improve the adsorption rate of antigen. The concentration of phosphate ions and the surface area of the aluminum hydroxide adjuvant grains are the factors that determine the amount of antigen adsorption. The addition of a phosphate group to the anionic form at PH = 7.4 can increase the negative charge on the surface without changing the type of adsorbed protein. Although some progress has been made in the modification of aluminum hydroxide adjuvant with phosphate ions, the addition of phosphate ions will inevitably cause a competitive pressure on the adsorption of antigen containing phosphate group and the aluminum hydroxide adjuvant, thus preventing it from playing a greater adsorption effect. So, looking for alternatives to phosphate ions for aluminum hydroxide adjuvant modification may be an effective research direction [94].

### 7.2. Nanochemical Prospects

As research on nanoparticles within the realm of adjuvant applications advances, the benefits of nanoparticle adjuvants, including excellent biocompatibility, immune enhancement, nano-size effects, antigen-loading capacity, sustained antigen release, and tissue targeting, have piqued the interest of researchers [95]. Nanoparticle adjuvants can provide a larger surface area, making the combination of the carrier and the antigen in the vaccine more effective [96]. The dimensions of adjuvants significantly influence immunogenicity. They can negatively impact the immune system, as nanoscale concentrations of adjuvants are highly immunogenic, eliciting a robust and enduring immune response. Smaller-sized adjuvants readily accumulate in tumors and lymph nodes [97].

Structural properties of nanoparticle carriers: (1) Surface effects: As the particle size decreases, the ratio of the number of surface atoms to the total number of atoms increases significantly. This change in ratio can alter the properties of the nanoparticles. (2) Small size effect: the reduced size of nanoparticles leads to unique behaviors that differ from larger particles. (3) Volume effect: Due to their minimal size, nanoparticles contain fewer atoms, which causes phenomena such as adsorption, catalysis, diffusion, and sintering to differ markedly from those observed in larger conventional materials [98]. These behaviors cannot be explained by the characteristics of bulk matter, which typically contains a vast number of atoms. This phenomenon is often referred to as the volume effect. (4) Quantum effect: Nanoparticles exist between atoms, molecules, and bulk solids, with energy levels in continuous materials becoming discrete at the nanoscale. As the particle size decreases, the spacing between the energy levels increases. When thermal, electric, or magnetic energy is smaller than the average energy level spacing, nanoparticles exhibit unique characteristics that are fundamentally different from those of macroscopic objects, known as the quantum effect. (5) Phantom number structures: Nanoparticles with sizes of less than 2 nm are often referred to as atomic clusters. These clusters become particularly stable when they contain a specific number of atoms, known as the magic number.

Nano-adjuvants are minuscule particles at the nanoscale that enhance stability, boost vaccine effectiveness, and elicit robust immune responses. Examples of nano-adjuvants include damage-associated molecular patterns (DAMPs) [99], pathogen-associated molecular patterns (PAMPs), aluminum, emulsions [100], saponin-based adjuvants, and Toll-like receptor (TLR) adjuvants. Furthermore, particle size may significantly influence their adjuvant efficacy, suggesting that nanoparticles could offer more advantages as vaccine adjuvants compared to microparticles [101]. For instance, the adjuvant dose can be lowered, thereby minimizing the side effects associated with aluminum-salt-based adjuvants, such as local irritation, adverse effects, and inflammatory responses [102]. However, nanoparticle aggregation can pose a challenge. Factors like the presence of salts and the pH of the dispersion medium can impact particle aggregation. To counteract this, stabilizers using electrostatic or steric mechanisms can be employed to prevent aggregation.

Gao [103] re-engineered the layered double hydroxide (LDH) NanoAlum. This novel NanoAlum not only retains the advantageous properties of AlOOH and Mg(OH)_2_ but also enhances its binding affinity with antigen-presenting cells (APCs). By optimizing its physical characteristics such as shape, particle size, and hydroxyl content, it targets and accumulates in lymph nodes, effectively improving the abnormal physicochemical properties of the tumor microenvironment and stimulating balanced humoral and cellular immune responses. Enhancements to the physical properties of aluminum adjuvants can be complemented by modifications to their surface groups, which have been shown to increase antigen loading and immunostimulatory potential [104]. Sun et al. [105] developed a surface-functionalized aluminum hydroxide variant, notably NH2-functionalized aluminum hydroxide nanorods, which exhibit elevated cell uptake, lysosomal disruption, oxidative stress induction, and NLRP3 inflammasome activation in mouse models. These findings underscore the significance of surface chemistry in optimizing adjuvant performance.

Overall, the uniformity of the nano-aluminum adjuvant surpasses that of traditional aluminum adjuvants [106]. The antigen it coats exhibits a strong affinity for macrophages and dendritic cells. Additionally, when the nano-adjuvant is conjugated with a polypeptide antigen or DNA vaccine, it avoids the carrier effect associated with conventional adjuvants [107], thus protecting the antigen and achieving an optimal immune response. Furthermore, the biocompatibility and biodegradability of the nano-aluminum adjuvant offer significant advantages by reducing potential cytotoxicity and enhancing patient safety. In preclinical trials, nano-aluminum adjuvants have demonstrated a remarkable ability to induce both humoral and cell-mediated immunity [108], suggesting their potential to elicit a comprehensive immune response. This dual action is particularly beneficial for vaccines targeting pathogens that require multifaceted immune defenses. Additionally, the controlled release properties of the nano-aluminum adjuvant ensure sustained antigen release, prolonging antigen exposure and enhancing immunogenicity. This feature is critical for vaccines targeting diseases that necessitate long-term immunity. The nano-formulation also facilitates targeted delivery to the lymph nodes, which are key sites for initiating immune responses. Recent advancements in nanotechnology have enabled the functionalization of nano-aluminum adjuvants with ligands or antibodies, further enhancing their specificity and efficacy. This opens up new possibilities for designing personalized vaccines tailored to individual patients’ immunological profiles. Moreover, the cost-effectiveness of manufacturing nano-aluminum adjuvants makes them a viable option for mass vaccination campaigns [109], especially in low-resource settings. The ability to produce these adjuvants consistently and at a lower cost without compromising quality is essential for global health initiatives.

As research progresses, it is imperative to conduct comprehensive clinical trials to fully understand the long-term safety and efficacy of nano-aluminum adjuvants. The integration of this advanced adjuvant technology into existing and new vaccines holds the promise of revolutionizing preventive medicine and effectively enhancing our capability to combat emerging infectious diseases.

## 8. Conclusions

After an extensive collection, reading, sorting, and analysis of the literature, it is clear that aluminum adjuvants have long been the most important vaccine adjuvants, playing an indispensable role in immunization [110]. Despite facing challenges from many emerging adjuvants, aluminum adjuvants continue to be widely used in clinical trials and vaccine development due to their excellent ability to enhance immune responses and their stable physical and chemical properties. Additionally, as research progresses, the mechanisms behind the action of aluminum adjuvants are gradually being unveiled. Moving forward, a key focus of future research will be to leverage the advantages of aluminum adjuvants while addressing their limitations.

## Figures and Tables

**Table 1:** FDA-approved aluminum adjuvant containing vaccines and their aluminum containing adjuvant (https://www.fda.gov/vaccines-blood-biologics/vaccines/vaccines-licensed-use-united-states, https://www.cdc.gov/vaccinesafety/concerns/adjuvants.html, accessed on 10 September 2024) (AAHS-amorphous aluminum hydroxyphosphate sulfate).

Vaccine	Trade Name	Manufacturer	Dose (Al^3+^)	Adjuvant
Anthrax Vaccine Adsorbed	Biotharax	Emergent BioSolutions	1.2 mg	Aluminum hydroxide
Tetanus and Diphtheria Toxoids Adsorbed	/	MassBiologics	0.53 mg	Aluminum phosphate
Diphtheria and Tetanus Toxoids Adsorbed	/	Sanofi Pasteur	0.33 mg	Aluminum phosphate
Tetanus and Diphtheria Toxoids Adsorbed	TENIVAC	Sanofi Pasteur	0.33 mg	Aluminum phosphate
Diphtheria and Tetanus Toxoids and Acellular Pertussis Vaccine Adsorbed	DAPTACEL	Sanofi Pasteur	0.33 mg	Aluminum phosphate
Tetanus Toxoid Reduced Diphtheria Toxoid and	Adacel	Sanofi Pasteur	0.33 mg	Aluminum phosphate
Diphtheria and Tetanus Toxoids and Acellular Pertussis Adsorbed and Inactivated Poliovirus Vaccine	Quadracel	Sanofi Pasteur	0.33 mg	Aluminum phosphate
Diphtheria and Tetanus Toxoids and Acellular	Pentacel	Sanofi Pasteur	0.33 mg	Aluminum phosphate
Diphtheria and Tetanus Toxoids and Acellular Pertussis Vaccine Adsorbed	INFANRIX	GSK	0.625 mg	Aluminum hydroxide
Tetanus Toxoid, Reduced Diphtheria Toxoid and Acellular Pertussis Vaccine, Adsorbed	Boostrix	GSK	0.39 mg	Aluminum hydroxide
Diphtheria and Tetanus Toxoids and Acellular	KINRIX	GSK	0.6 mg	Aluminum hydroxide
Diphtheria and Tetanus Toxoids and Acellular	Pediarix	GSK	0.85 mg	Aluminum hydroxide
Human Papillomavirus Bivalent (Types 16 and 18) Vaccine, Recombinant	Cervarix	GSK	0.5 mg	Aluminum hydroxide + monophosphoryl lipid A
Human Papillomavirus Quadrivalent (Types 6, 11, 16 and 18) Vaccine, Recombinant	Gardasil	Merck	0.225 mg	AAHS
Hepatitis A Vaccine, Inactivated	VAQTA	Merck	0.225 mg	Ammorphous Hydroxyphosphate Alumulphate
Haemophilus influenza B	PedVaxHIB	Merck	0.225 mg	AAHS
Meningococcal Group B Vaccine	BEXSERO	GSK	0.519 mg	Aluminum hydroxide
Meningococcal Group B Vaccine	TRUMENA	Pfizer	0.25 mg	Aluminum phosphate
Pneumococcal 13-valent Conjugate Vaccine	Prevnar 13	Pfizer	0.125 mg	Aluminum phosphate
Hepatitis B	Recombivax HB	Merck	0.5 mg (adult)	AAHS

**Table 2:** Comparison of aluminum content standards of aluminum-containing adjuvant vaccines (Chinese Pharmacopoeia 2020, 4 Edition).

Vaccine Varieties	Types	Contents
Adsorbed pertussis diphtheria combined vaccine	Aluminum hydroxide	0.17–0.26 mg/dose
Hepatitis A inactivated vaccine (human diploid cell)	Aluminum hydroxide	0.35–0.62 mg/mL
Adsorbed cell-free pertussis combined vaccine	Aluminum hydroxide	0.17–0.26 mg/dose
Adsorbed diphtheria–tetanus combined vaccine (for adults and adolescents)	Aluminum hydroxide	≤0.43 mg/dose
Adsorbed diphtheria vaccine	Aluminum hydroxide	≤0.52 mg/dose
Adsorbed tetanus vaccine	Aluminum hydroxide	≤0.52 mg/dose
Recombinant hepatitis B vaccine (CHO cells)	Aluminum hydroxide	≤0.43 mg/mL
Bivalent hemorrhagic fever with renal syndrome inactivated vaccine (hamster kidney cells)	Aluminum hydroxide	≤0.24 mg/mL
Recombinant hepatitis B vaccine (Hansenula yeast)	Aluminum hydroxide	≤0.62 mg/mL
Tick-borne encephalitis vaccine	Aluminum hydroxide	≤0.24 mg/mL
Recombinant hepatitis B vaccine (brewing yeast)	Aluminum phosphate	≤0.62 mg/mL
